# Marjolin Ulcer: Squamous Cell Carcinoma Arising in a Modeling Agent-Induced Ulcer

**DOI:** 10.7759/cureus.93740

**Published:** 2025-10-02

**Authors:** Naomi H Domínguez-Wakida, Azyadeth Gracián-Castro, Marcela Hernández-Vera, Ilse Y Osorio-Aragón, Elisa Vega-Memije

**Affiliations:** 1 Dermatology, Hospital General "Dr. Manuel Gea González", Mexico City, MEX; 2 Dermatopathology, Hospital General "Dr. Manuel Gea González", Mexico City, MEX

**Keywords:** malignant transformation, marjolin ulcer, mineral oil, modeling agent reactions, squamous cell carcinoma

## Abstract

The use of modeling substances, such as mineral oil, can trigger chronic inflammatory reactions with serious complications, including persistent ulceration. In rare cases, these lesions may undergo malignant transformation to aggressive cutaneous neoplasms, such as Marjolin ulcers. We present the case of a 55-year-old transgender woman with a history of mineral oil injection who developed a chronic leg ulcer that underwent malignant transformation, with histological confirmation of a well-differentiated squamous cell carcinoma. This case highlights the importance of close monitoring of chronic ulcers in this patient population and underscores the severe consequences of using unregulated modeling agents.

## Introduction

The use of unregulated modeling substances, such as mineral oil, has become a common but high-risk aesthetic practice. These substances can induce chronic inflammatory reactions that lead to serious complications such as fibrosis, ulceration, and necrosis [1]. In the context of persistent inflammation, there is a risk of malignant transformation, most notably the development of a Marjolin ulcer. This entity refers to an aggressive cutaneous malignancy that arises in previously damaged or chronically injured skin, most commonly in burn scars but also in traumatic wounds or chronic ulcers [2,3]. While squamous cell carcinoma is the most frequent histological type, other variants such as basal cell carcinoma and melanoma have also been reported. Although rare, Marjolin ulcers account for up to 2% of cutaneous squamous cell carcinomas and are associated with higher rates of metastasis [4]. Malignant transformation typically occurs after an average of 30 years from the initial lesion [5]. This case illustrates the consequences of using unregulated materials and highlights the importance of clinical vigilance in the presence of long-standing skin lesions.

## Case presentation

**Figure 1:**
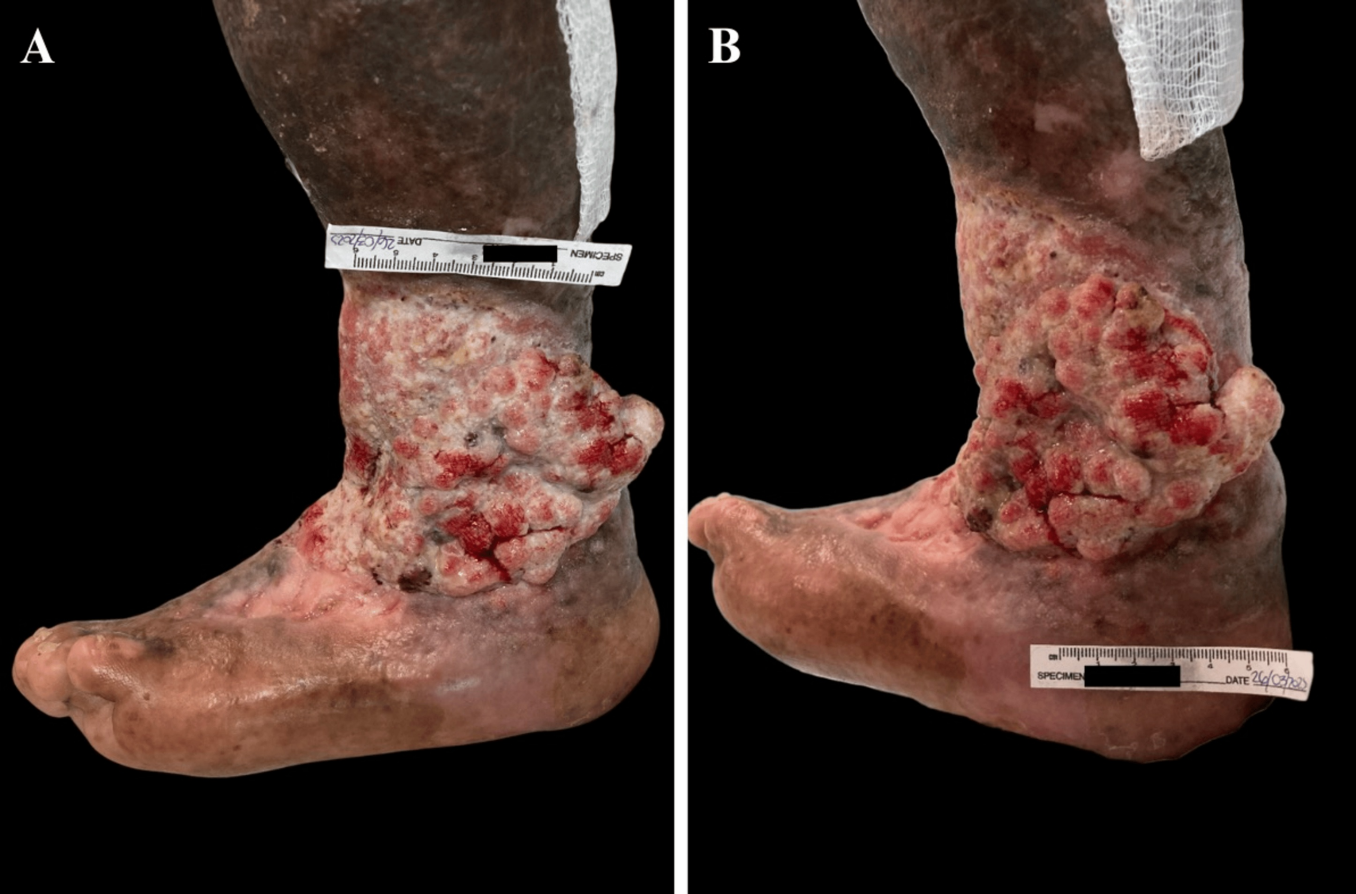
Evolution of the ulcer towards a tumor-like appearance. (A-B) Clinical appearance of the wound two years after the last evaluation. The wound bed is notably hypergranular and exophytic.

A 55-year-old transgender woman presented to our service with a growth on her left leg, arising from a 22-year-old chronic ulcer. Physical examination revealed a tumor-like lesion localized to the lower left extremity affecting the distal third of the leg at the level of the medial and lateral malleoli and anterior surface. The lesion was characterized by an 18 x 10 cm exophytic ulcer with a verrucous surface, friable tissue that bled on contact, and abundant fibrin covering the ulcer bed. The edges were indurated and rolled, and the surrounding skin showed a sclerodermiform appearance with areas of ochre-colored dermatosis (Figures 1A-1B).

**Figure 2:**
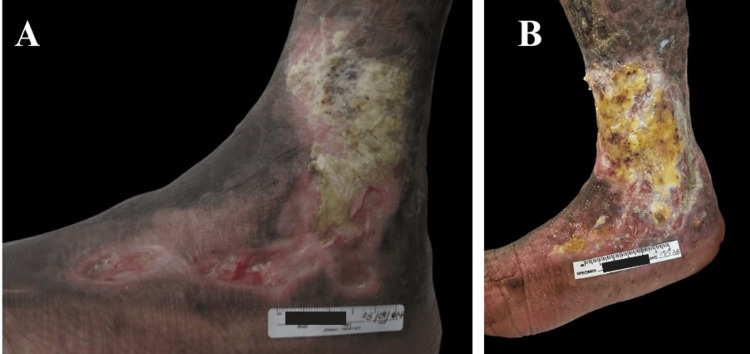
Stationary wound improvement. (A) Initial presentation of the wound. (B) Clinical evolution over a nine-year treatment period, showing an increase in ulcer size before the patient was lost to follow-up.

During the medical history interview, the patient reported mineral oil injections in the buttocks in 1986. Seventeen years after the injection of the modeling material, the patient developed ulcers on both buttocks, which required surgical washouts with adequate wound re-epithelialization. Ten years later, ulcers appeared on both lower extremities at the malleolar level. The patient was treated at our wound care clinic with advanced wound management and multiple courses of treatment for soft tissue infections, with stationary wound progression (Figures 2A-2B). The patient had poor adherence to treatment, leading to a worsening of the lesions, and was eventually lost to follow-up after nine years of care. She returned in April 2025 due to worsening pain and malodor from the skin ulcers.

**Figure 3:**
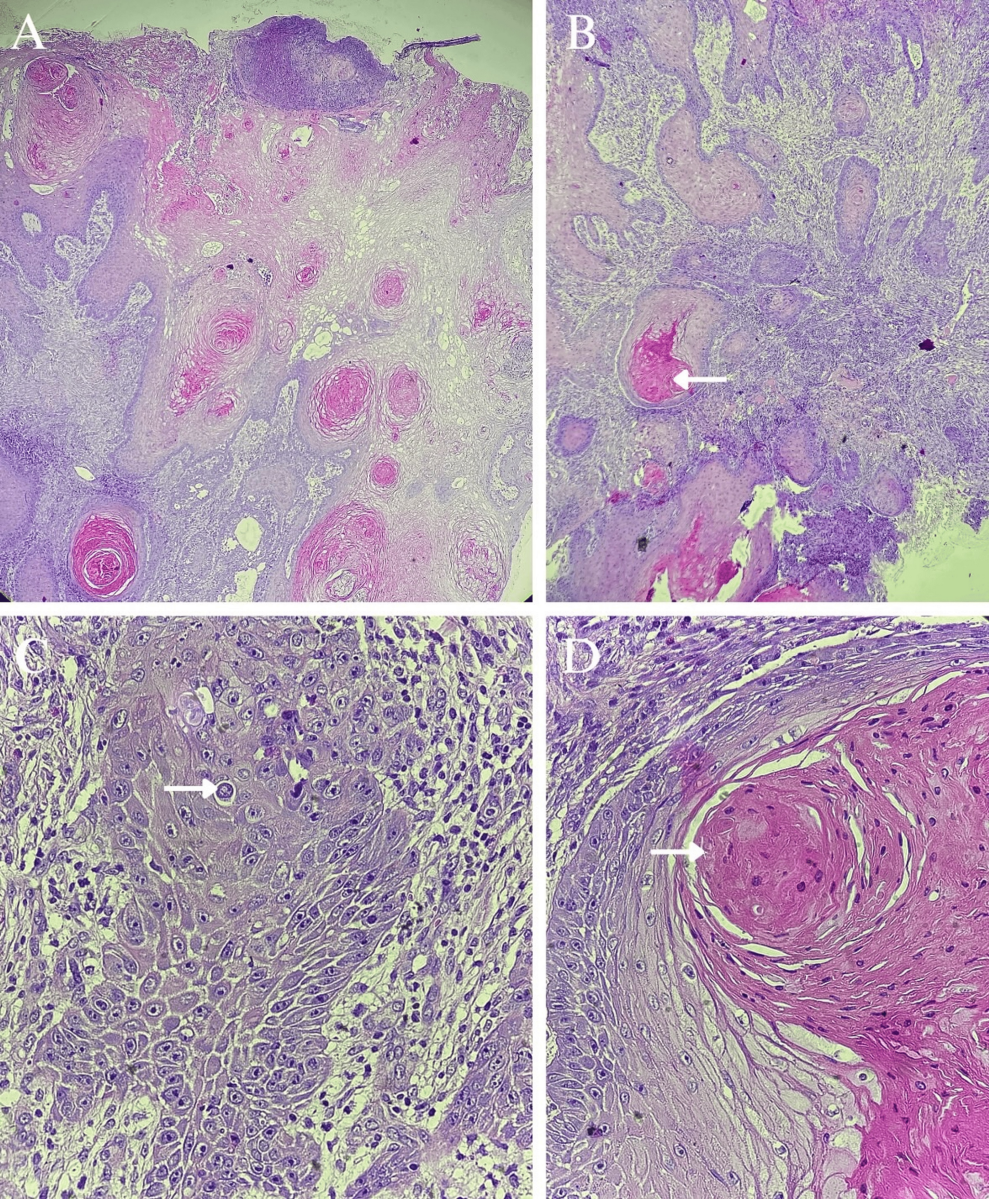
Histological findings. (A) Ulcerated epithelial neoplasm arranged in cords and lobules of varying sizes (H&E, 4X). (B-C) Tumor composed of cells with abundant eosinophilic cytoplasm, nuclear pleomorphism (arrow), individual necrosis, atypical mitoses, and keratin pearls (arrow) (H&E, 10X and 40X). (D) Squamous eddy formation (arrow) (H&E, 40X).

Given the clinical characteristics of the lesion, a biopsy was performed with histopathological mapping by taking multiple punch biopsies from the ulcer. The biopsy revealed findings consistent with a well-differentiated, invasive, ulcerated squamous cell carcinoma (Figures 3A-3D).

**Figure 4:**
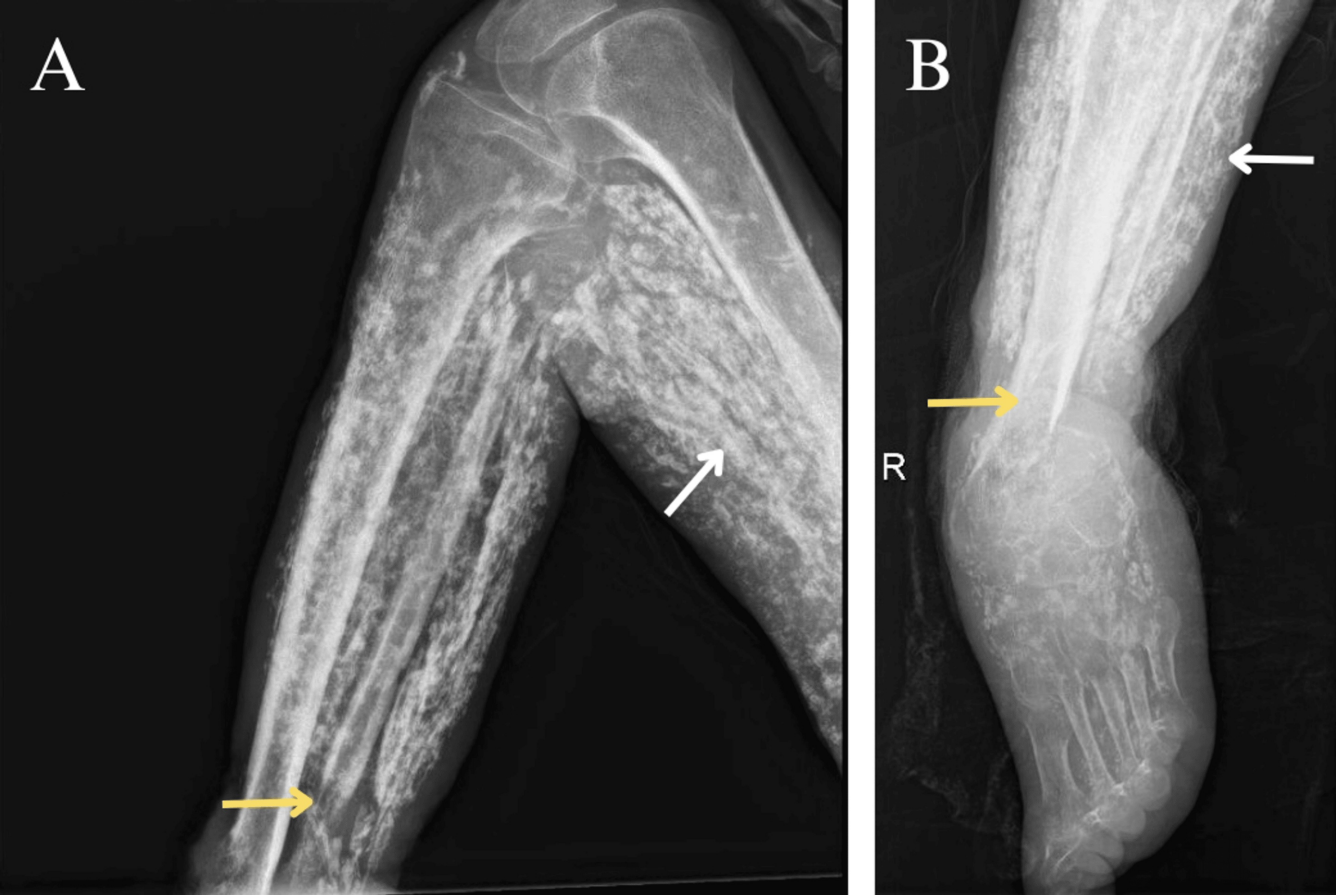
Radiological findings of the left lower limb. (A-B) The findings show infiltration by exogenous modeling material (white arrows) and a comminuted, displaced fracture of the distal tibia and fibula (yellow arrows).

The patient was referred to the oncology department, where staging studies were performed. Radiographs of the left leg revealed infiltration of the modeling material and a pathological fracture (Figures 4A-4B). Computed tomography (CT) excluded distant metastases, and curative-intent surgery was planned, resulting in a left above-knee amputation. Based on the multidisciplinary evaluation, adjuvant pharmacological therapy was deemed unnecessary. The patient remains under clinical and imaging surveillance at our center, with no evidence of local recurrence or systemic progression to date.

## Discussion

Foreign modeling agent reactions (FMARs) occur following the injection of high-viscosity substances for cosmetic purposes, leading to severe complications such as ulceration, disfigurement, and even death. These practices have reached epidemic proportions in some regions worldwide. The earliest documented cosmetic use involved paraffin. Subsequent reports have described late-onset reactions including material migration, ulceration, fistulas, infections, necrosis, inflammatory granulomas, pulmonary embolism, and even death. While initially applied exclusively by plastic surgeons, these substances are now frequently administered by non-medical personnel. Reported materials include paraffin, silicone, various oils (mineral, vegetable, motor), and other agents such as lanolin, beeswax, animal fat, and synthetic polymers. In Mexico City, the most commonly used are mineral oil, vegetable oil, guayacol, and silicone [1].

Due to their fluidity, these materials can migrate by gravity or lymphatic pathways, causing distant lesions. The most affected areas include the buttocks (38.5%), legs, thighs, breasts, face, back, and abdomen. Common local clinical findings include inflammation, woody fibrosis, scarring, pigmentary alterations, necrosis, ulceration, fistulas, and exposure of the injected material. Treatment is often prolonged and complex, potentially resulting in chronic ulcers [1].

Although local inflammatory reactions to modeling agents are the most frequent manifestation, this case documents a far more severe complication: malignant transformation with clinical and histological features consistent with a Marjolin ulcer. The incidence of malignancy arising from such reactions has not yet been established in the literature.

Marjolin ulcers are aggressive cutaneous neoplasms that develop on previously damaged or chronically injured skin, most commonly in burn scars, and account for up to 2% of cutaneous squamous cell carcinomas [2-4]. Malignant transformation may be related to reduced blood flow in the affected area, along with a loss of immune surveillance due to the depletion of immune cells in chronic scars. This allows tumor cells to escape immune control and adopt a more aggressive behavior with increased metastatic potential. Chronic irritation further promotes cell proliferation, thereby increasing the risk of mutations [2,5].

Although burn scars are the most frequently reported predisposing factor, Marjolin ulcers can also arise in traumatic wounds, venous stasis ulcers, osteomyelitis, pressure ulcers, radiation dermatitis, insect bites, animal bites, or hidradenitis suppurativa [2,4].

These lesions typically affect individuals in their fifth decade of life. Men are twice as likely to develop this condition compared to women, possibly due to a higher incidence of burns in this demographic group [2,4,5]. The average latency period for malignant transformation is approximately 30 years, though it can range from 5 to 50 years. Based on this latency, Marjolin ulcers are classified as acute or chronic; in the acute form, malignant transformation occurs within the first 12 months after the initial injury, and basal cell carcinoma is more frequently observed in these cases [2,4-6].

From a clinical standpoint, patients typically report a longstanding wound or scar that fails to heal properly and has recently undergone changes such as ulceration, pain, bleeding, exudate, or progressive exophytic growth. The lower extremity is the most commonly affected site, followed by the scalp, upper extremities, trunk, and face [2,4]. The most frequent clinical presentation is an ulcerated, flat lesion with raised edges and peripheral induration. Two main morphological variants have been described: a well-differentiated exophytic form, generally associated with a better prognosis, and a poorly differentiated ulcerative form, which carries a worse prognosis due to its invasive behavior [4].

Malignant transformation should be suspected in chronic ulcers persisting for more than three months, particularly if they exhibit irregular growth, raised or rolled borders, persistent ulceration, and a foul odor. Additional warning signs include exophytic granulation tissue, spontaneous bleeding, poor response to standard treatments, and the presence of regional lymphadenopathy [2,4].

Any chronic lesion or scar that shows changes should raise a high index of suspicion and prompt biopsy, preferably via excision, incisional technique, or punch biopsy [2,4]. Definitive diagnosis is established through histopathological examination. Most cases correspond to squamous cell carcinoma (80-90%), though basal cell carcinomas and melanomas have also been reported. In acute forms, basal cell carcinoma predominates [5]. Unlike primary carcinomas, Marjolin ulcers exhibit significantly higher rates of metastasis (30-40%) and recurrence, compared to 0.5-3% observed in conventional squamous cell carcinoma [2,6]. Histologically, squamous cell carcinoma is characterized by atypical squamous cells with frequent mitotic figures [2]. Imaging studies such as X-rays, CT, or magnetic resonance imaging (MRI) are recommended to assess local extension and detect metastasis [2,4].

Proper management of chronic wounds remains the most effective strategy to prevent the development of Marjolin ulcers. The most widely accepted curative treatment is wide excision with 2-3 cm margins; when this is not feasible, grafts or flaps may be employed. Amputation is reserved for cases with bone involvement or when complete resection is not possible. Radiotherapy and chemotherapy are considered in cases with poor prognostic factors or metastatic disease. Lymph node evaluation via ultrasound and sentinel lymph node biopsy may help guide surgical management. Despite treatment, recurrence may reach up to 50%, and mortality approaches 21%, with histologic grade and lymph node involvement being the main prognostic factors [2,4,5,7].

There are a few reports in the medical literature describing malignant transformation secondary to modeling agents or implants. In 2000, a case of penile squamous cell carcinoma associated with mineral oil injection was described [8]. In 2022, Toyonaka et al. reported a case of squamous cell carcinoma developing 16 years after silicone breast injection [9]. In 2008, Chalmers et al. reported multiple digital squamous cell carcinomas related to occupational exposure to soluble oil (Table 1) [10]. While complications from unregulated modeling substances are relatively common, malignant transformation remains rare and is limited to anecdotal reports without an established incidence, underscoring the relevance of the present case.

**Table 1:** Comparative table of reported cases in the literature

Authors	Ciancio and Coburn [8]	Toyonaka et al. [9]	Chalmers et al. [10]	Our case
Sex	Male	Female	Male	Male
Age	55 years	52 years	77 years	55 years
Topography	Penis	Left breast	Both hands (mainly thumbs) and the penis	Left leg
Clinical findings	Ulcerated mass, 15 cm	Suppurative ulcerated mass	Verrucous and ulcerated masses	Verrucous ulcerated mass, 18 × 10 cm
Evolution of ulcer	Not specified	Not specified	Not specified	22 years
Substance exposure	Mineral oil injection in the penis	Liquid silicone injection in both breasts	Worker exposed to mineral oils	Mineral oil injection in the buttocks
Latency	35 years	16 years	50 years of exposure	39 years
CT findings	No pelvic adenopathy; calcified granulomas in both lungs and abdomen	Multiple calcified subcutaneous nodules in the breasts	-	Distant metastasis excluded
Histopathological diagnosis	Squamous cell carcinoma	Well-differentiated squamous cell carcinoma	Invasive squamous cell carcinoma and erythroplasia of Queyrat	Well-differentiated invasive squamous cell carcinoma
Treatment	Surgical excision with margins and split-thickness skin graft	Total mastectomy, lymph node dissection*, and chemotherapy	Surgical excision	Limb amputation
* Sentinel lymph node biopsy: positive for one-step nucleic acid amplification, but histopathology was negative for nodal metastasis.				

## Conclusions

This case underscores the need for careful monitoring of chronic ulcers, particularly in patients with a history of foreign modeling agent injections. These practices, beyond their destructive local effects, may be associated with serious complications, including malignant transformation. Prevention, early diagnosis, and close follow-up are essential to avoid such adverse outcomes.
